# Resilience: Conceptualization and Keys to Its Promotion in Educational Centers

**DOI:** 10.3390/children9081183

**Published:** 2022-08-07

**Authors:** Isaac Moll Riquelme, Sara Bagur Pons, Maria Rosa Rosselló Ramon

**Affiliations:** 1 Department of Applied Pedagogy and Educational Psychology, Faculty of Education, University of the Balearic Islands, 07730 Alaior, Spain; 2 Department of Applied Pedagogy and Educational Psychology, Faculty of Education, University of the Balearic Islands, 07122 Palma, Spain

**Keywords:** resilience, school, inclusion, psychological needs, teacher role

## Abstract

This article conceptualizes resilience, a capacity present in all people that can be activated to face and overcome adversity. Based on a systematic review of the literature, the different lines by which research on this subject has been developed to the present moment, when it is interpreted from neuroscience, are identified. From the first descriptions of risk and protective factors, the concept has moved through the consideration of resilience as a process that can be carried out by anyone and not as an intrinsic characteristic of exceptional people, to the study of the will to not let oneself be discouraged and the personal commitment to overcome. Next, the keys to promote this capacity in educational centers are analyzed. There are two main focuses of intervention: the relationship that the teacher can establish with students and the pedagogical strategies and resources that can be used. From the purpose of the integral formation of people and in an inclusive framework, educational centers have the challenge and responsibility to promote resilient processes in all students, especially in those who experience more barriers to learning and participation due to personal circumstances and adversities. In this way, educational centers are encouraged to be protective environments where all students feel safe and can empower themselves and unfold their potential. The review has been conducted following the Preferred Reporting Items for Systematic Reviews and Meta-Analyses (PRISMA) protocol, and the bibliographic search has been performed in the Dialnet Plus, Web of Science (WoS) and Scopus databases.

## 1. Introduction

Resilience is understood as a person’s capacity to respond to adversities, as well as to overcome and emerge stronger from them [1,2]. According to [3], resilience is a highly dynamic process of positive adaptation where, according to [4], there is an interaction between internal and external factors that allows difficulties to be overcome constructively.

In the socio-educational field, the resilience of students should be considered, especially those who may present socio-emotional difficulties. It should be noted that resilience is a multidimensional phenomenon, since it implies taking into account individual, family and sociocultural contextual factors, from which protective factors and risk factors are derived [5]. Specifically, risk factors in the child population are identified, among others, in an overload of responsibilities, physical and psychological abuse, marginalization, poverty, parental mental illness or impoverished parenting practices [6,7]. Protective factors are those that allow the child to overcome adversities [8], generating possibilities for successful coping [9], classified into personal, family, psychosocial and sociocultural factors [10].

The school is one of the contexts where children will spend more time and should provide an environment in which everyone can feel safe, facilitating their emotional development, in addition to gaining other competencies and mastering curricular content. Given that resilience is not an innate capacity of the person, but a learned one [11], the teacher has an important role in increasing this capacity, creating ideal conditions for learning to occur while enhancing protective factors in the classroom coexistence environments [12].

Children with some educational needs may have difficulties in “*relating to peers, accepting limits and tolerating the frustration of not knowing, maintaining interest during the task, looking for strategies, planning, accepting mistakes and correcting them, finishing tasks, advancing and working with some autonomy*” [13] (p. 35). In this way, education has to constitute a protective environment that acts in favor of resilience and helps children’s emotional difficulties not to end up causing them to fail at school. In short, it must play a preventive role in the face of possible situations of social exclusion.

This article aims to deepen the knowledge of those factors that facilitate the school to be a promoter of resilience for all students, thus contributing to school and social inclusion. The characteristics of the relationship between the teacher and the child that can promote resilience are presented, as well as some pedagogical strategies and resources that can be used to encourage classrooms and schools to be safe and optimal spaces for development and learning.

## 2. Materials and Methods

### 2.1. Search Strategies and Selection of Studies

The methodology used was a systematic review of the literature, following the Preferred Reporting Items for Systematic Reviews and Meta-Analyses (PRISMA) protocol, which promotes research quality [14], in addition to the possibility of replicating the process followed [15]. Specifically, a bibliographic search was conducted in Dialnet Plus, Web of Science (WoS) and Scopus, as highly recognized databases associated with the social sciences. The selection criteria were year of publications, language of publication, field of study and location of keywords (Table 1).

Table 2 presents the initial results obtained through the search equations, formed by the keywords and their synonyms in each database.

Through an initial reading of the titles and summaries of the documents, a first screening of the documents was carried out according to their suitability for the objectives of this study.

### 2.2. Inclusion and Exclusion Criteria

The inclusion criteria were as follows: the promotion of resilience took place in educational centers (1), the framework for action was inclusive (2), the study of resilience was focused on children with emotional needs derived from affective attachments in childhood or other adverse events (3).

The exclusion criteria were as follows: the promotion of resilience was focused on non-child agents (1), resilience building was focused on specific curricular areas (2).

**Figure 1:**
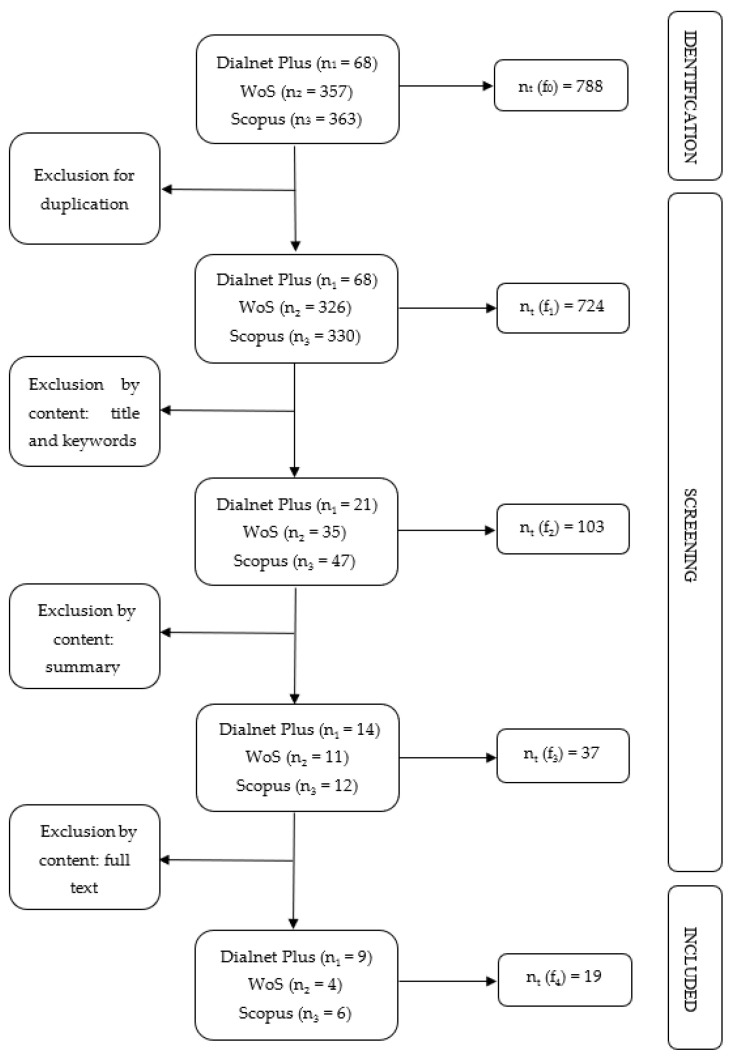
Flow chart of the selection process. Own elaboration based on PRISMA protocol.

Thus, the flow chart on the inclusion process is presented below (Figure 1).

The database search found 788 initial papers that met the selection requirements. After discarding duplicates, 724 documents remained. Taking into account the inclusion and exclusion criteria indicated above, 103 references were left out after reading the titles and keywords. The same criteria were applied to select the remaining documents based on the content of the abstract. Finally, 37 references were subjected to full-text analysis applying the same criteria, leaving 19 resulting documents (Table 3): 11 articles (57.9%), 5 books (26.3%), 2 chapters (10.5%) and 1 thesis (5.3%).

### 2.3. Data Analysis

The selected documents were read by the researchers and their content was grouped into different categories. The resulting categories were: lines of research on resilience (C1), resilience and neuroscience (C2) and promoting resilience in schools (C3). In turn, category 3 was divided into two subcategories: the relationship between the teacher and the student (S1C3) and classroom and school climate (S2C3).

## 3. Results

### 3.1. Lines of Resilience Research and the Evolution of the Field of Study

The term resilience comes from the Latin *resiliens*, participle of the verb *resilire*, which means to jump back, to bounce back. Initially, this word was used in the field of physics, specifically in the study of the properties of materials, to describe their ability to return to their original shape after having been subjected to deformation. The introduction of this term in the field of psychology took place later and began in Anglo-Saxon countries. In Spain, the use of the concept in the field of human sciences has been more recent. In fact, until the 23rd edition of the Dictionary of the Spanish Language [16], the entry “resilience” did not appear.

Following [17], the studies reviewed can be classified into three lines of research that coincide, in turn, with different temporal stages: in the first, efforts initially focused on identifying and analysing the personal characteristics of individuals who seemed immune to adversity. Later, still in the same stage, the factors present in the environment that could help explain this supposed immunity were also studied.

In the second line of research, which is more educational in nature, resilience began to be conceived more as a process than as a state, and research focused on explaining how people could become resilient based on the interaction between their personal characteristics, the protective elements present in the environment and the experience of the adverse situation itself. In the third line, research was conducted on the internal energy, present in all people, which drives self-actualization. At present, all the elements on which the different lines of research on this phenomenon have focused their attention are taken into account. Resilience is conceived as a process that the person activates to face adversity, which is nourished by personal abilities and skills, by the protective factors of the environment, and which allows him/her to achieve new competencies to project him/herself positively in the future.

#### 3.1.1. First Line: Protective Factors

To this first line belong the studies that focused on identifying the qualities possessed by people who seemed immune to the risk factors to which they were subjected. Resilience was considered as an “*individual capacity based on certain personal characteristics*” [18] (p. 29). In the same sense, Kreisler (1996, cited in [19]) defines resilience as “*the capacity of a subject to overcome circumstances of special difficulty, thanks to his or her mental, behavioral and adaptive qualities*” (p. 25).

As progress was made in identifying these personal qualities, the focus of interest broadened and other factors began to be detected which, despite not being part of the personal characteristics of individuals, were present in their immediate environment and also seemed to protect them from the different risk factors that threatened them. We began to speak of protective factors and the presence of these elements was linked to the fact that a person resisted and overcame the experience of certain adverse experiences.

Protective factors are defined as “*characteristics of the person or the environment that mitigate the negative impact of stressful situations and conditions*” [20] (p. 27). According to [21], protective factors can be found in the individual, the family and the social environment. The main protective factors identified in the studies reviewed are shown in Table 4.

In this line of identification of protective factors, other authors [27,28,29,30] have defined what they have described as pillars of resilience or dimensions existing in resilient people (Table 5), which have much to do, above all, with the individual’s own qualities. Although resilience is not currently understood exclusively from the point of view of having a set of qualities, but is explained as a process that is being built, as explained in [31], there are individual characteristics of the person that positively influence this process. Moreover, these qualities can be acquired through the resilience process.

#### 3.1.2. Second Line: Resilience as a Process

Having identified the personal characteristics of resilient individuals and the protective factors that the environment could offer, the interest of researchers focused on finding out whether personal qualities were innate and were or were not present, or whether they could be developed through the environment. Resilience began to be conceived from a more dynamic point of view, understanding that “*the person, rather than “being” resilient, “is” resilient*” [32] (p. 42), or as [31] put it, “*from understanding resilience as a quality or an adjective, to understanding it as a process or an action*” (p. 221). Resilience began to be considered a “*process that leads individuals, groups or communities to overcome adversity*” [18] (p. 29). Along the same lines, [33] states that:

*To speak of resilience in terms of the individual is a fundamental error. One is not more or less resilient, as if one possessed a catalog of qualities: innate intelligence, resistance to pain, or the molecule of humor. Resilience is a process, a becoming of the child who, by dint of actions and words, inscribes his development in an environment and writes his history in a culture. Consequently, it is not so much the child who is resilient as its evolution and its process of vertebration of its own history.*

(p. 214)

It is understood that “*resilience is modifiable, it is not static*” [25] (p. 95) and, therefore, it depends on the interaction of the individual with the elements of his or her immediate environment, among others with the people who are part of it. The works of [34], who define resilience as a “*set of social and intrapsychic processes that take place over time and according to how the attributes of the child, the family and the social and cultural environments are combined*” (p. 233), and those of [3], who also understand resilience as a dynamic process that implies positive adaptation in a context of great adversity, belong to this current.

Based on the conception of resilience as a process in which individuals can build strengths by overcoming adversities and the importance of the environment and bonds with others in this process, the possibility of promoting resilience in people is beginning to be considered.

In this way, resilience ceases to be considered a characteristic of exceptional individuals with specific biological and psychological characteristics [32] and is understood as a process of change, growth and improvement that can be carried out by any person. Along these lines, [22] understands resilience as a “universal capacity” (p. 3) that every person can develop at any stage of his or her life cycle.

It is important to note that resilient behavior is not considered as a return to the state of well-being prior to the adverse event, but implies a process of personal growth: “it is not about returning to the same point” [26] (p. 36), but “*resilience leads to a metamorphosis of the individual*” [35] (p. 53). Following this reasoning, [36] state that it is not possible to return to the initial state and understand that “*resilience means generating options to metamorphose and continue living*” (p. 66).

#### 3.1.3. Third Line: The Will to Resurgence

The third line of research considers resilience as “the force that drives a person to grow through adversity” [17] (p. 307). It is understood that, although there are factors that favor the resilient process and that the people in the environment can be key elements, the individual’s will to not let himself be discouraged and the motivation to find meaning in what he is living and to make himself purposeful are also fundamental.

In this line, [32] expresses that:

*The person has a more active role not as the possessor of specific characteristics, but as the subject of his or her own history that he or she weaves, necessarily, within his or her social and cultural context. From this vision of resilience is transferred the power of choice in the face of adversity, consisting in not letting oneself be defeated, in divesting oneself of the condition of victim*.

(p. 33)

Continuing with this reasoning, [29] states that “*the most important thing is to want to, to accept the personal commitment to improve oneself*” (p. 158). And he adds that, in order to change, new neural circuits have to be created and that this implies putting into practice new ways of thinking, feeling and acting. “*The mind can be programmed in order to modify perception and, as a consequence, also vary the physiological and emotional response to change behavior*” [29] (p. 159). This last statement introduces us to the following section which shows how the resilience process can be explained from neuroscience.

### 3.2. Resilience and Neuroscience

In recent years, neuroscience has shown a growing interest in understanding how people learn, unlearn and relearn, and can generate changes in the way they interpret reality, behave and activate adaptive and resilient responses to adversity. There are three fundamental discoveries that form the basis on which the explanation of the resilient response from neuroscience is based: the production of new neurons (neurogenesis) throughout the life cycle, neuronal plasticity (neuroplasticity) and epigenetics. These discoveries open the door to being able to direct our own process of change and, consequently, our process of resilience:

We now know that people are not predetermined to live a life designed only by genetics. Biology is not a destiny because the mind also influences the brain and convinces it to lend all its resources (neurons and synapses) to being able to change, so that people can improve. New research in resilience experiments thus considers how certain lifestyles can influence the configuration of the brain. [36] (pp. 28–29)

On the one hand, we know that “*we have the capacity to continue making neurons in different structures of our brain and throughout our life. Although when we reach adulthood, we do not generate as much neurogenesis as when we are children, we continue to produce neurons*” [37] (p. 71).

On the other hand, we also know that “*neurons that are stimulated together are structured together, and neurons that are stimulated without being synchronized lose their mutual connection*.” [37] (p. 70). This is neuroplasticity, which can be positive or negative. As [37] explains, positive neuroplasticity involves creating new learning or adding information to our previous knowledge. In contrast, negative neuroplasticity involves the deconfiguration of a network, of a learning. Thanks to the plastic capacity of our brain “*we have the possibility of learning and making changes in the way we feel and behave*” [38] (p. 138).

Finally, epigenetics “*defines which parts of the genome potential are expressed and which are not (…) without genetic changes, there are biochemical changes (…) and these determine that some genes can be expressed and others silenced*” [39] (p. 71).

Thus, thanks to the capacity to produce new neurons, to be able to connect them by configuring new learning and to be able to influence, through new learning and habits, the expression and silencing of certain genes, we have the power and the responsibility to modify thinking habits to promote a more adaptive interpretation of reality and behavior that generates greater well-being.

*Behind every thought and every behavior there is a brain wiring responsible. Our behavior is a consequence of how our brain works and this is not only determined by our genome. Today we know the great importance of the influence of the environment and the attitude towards it in determining our brain wiring*.

[38] (p. 137)

Considering the different lines of research on resilience, the way in which the understanding of this phenomenon has evolved and the contributions of neuroscience, it can be affirmed that:

- The resilient response is an idiosyncratic process that every person can develop at any time in his or her life cycle, based on the willingness to activate and use his or her personal abilities and skills, the support of significant people and the use of other environmental resources.
- The resilient process, like all learning, generates a restructuring of neural networks that is possible thanks to the production of new neurons and brain plasticity. This possibility of modification makes it possible to learn new adaptive skills, unlearn other maladaptive ones and develop new habits of thought and behavior that can affect the expression or silencing of certain genes.
- This process, which can be activated in the face of adversity, implies a positive evolution of the person, which develops and/or reinforces his or her competencies and projects him or her towards the future with greater self-confidence to face new challenges.

### 3.3. Promoting Resilience in Schools

Schools have great potential to promote resilience in students. As [20] point out: “*After the family, the school is the most conducive place for students to experience the conditions that promote resilience*” (p. 37).

From the field of public health promotion, given the evidence that the perceived quality of life and that many diseases, both physical and mental, show an educational gradient, [40] are committed to “*moving from a model focused on risk to a model focused on prevention, taking people’s potentialities as a basis*” (p. 214). These authors advocate promoting health habits at school from a resilience-centered approach.

*The school, understood as a center of health and socialization, is a potential space to promote resilient people (…) the emphasis of public policies should focus on the development of human capabilities, that is, on an education for life*.

[40] (p. 214)

On the other hand, as [41] indicates, several researches conclude that depressive symptoms, recurrent negative thoughts and beliefs, and problems in the family environment exacerbate the low efficiency in the use of one’s own resources and the creation of habits to cope with academic activity. According to this author, promoting certain resilience factors such as social skills or self-confidence improves the emotional state of students and has a positive impact on their academic development.

Thus, the school is a privileged place to promote resilience, creating the conditions that allow for greater equity and educational quality for all. Promoting resilience at school is a way to promote inclusive education, since many of the children who, due to the circumstances in which they have lived or are living, would be heading towards a situation of school failure, can find understanding, affection, stability, encouragement, limits, can develop certain skills and create expectations that help them to project themselves into the future with confidence and hope.

In the same way that in the family context the attachment figure provides security for the child to explore the environment, in the educational context the teacher provides security for the student in the task of learning. With all children, but especially with those who have not found a secure environment in the family environment and have developed emotional needs, the school can act as a secure base to help them to emerge and maintain the desire to learn and to influence the modification of the modalities of relationship with themselves and with others [13].

Resilience bets on the capacity of every person to face and emerge stronger from adversities and, in order to do so, it is very important to be able to create a meaning to what is being lived. In this creation of meaning, the other person is very important as a person who accepts and welcomes the individual unconditionally. As [42] state: “*Acceptance will be a transversal axis in this creation of meaning from different resilient therapeutic proposals*” (p. 47). For these authors, resilience is built under the gaze of a resilience tutor who recognizes the other in a process of reconstruction of the value and dignity of the person. The concept of resilience coach was introduced by Cyrulnik, who described its importance as follows:

*(…) the encounter with a significant person. Sometimes it is enough with one, a teacher who with a phrase gave hope back to the child, a sports instructor who made him understand that human relationships could be easy, a priest who transfigured suffering into transcendence, a gardener, a comedian, a writer, anyone could give body to the simple meaning: “It is possible to come out successful”*.

[33] (p. 214)

In the educational center, the teacher can become that reference person for the students, especially for those who suffer emotionally, a person who understands them, accepts them as they are and stimulates them to find themselves again and to create a meaning to what they are living. The teacher can be someone who will provide the student with strategies that will empower him and allow him to develop in his environment. Following [43]:

*The role of the educator (…) beyond delivering a set of knowledge that exists by itself, must promote the construction of this knowledge to encourage the empowerment of the student. His mission is to help the student discover his potential so that he can build his own life project based on autonomous and responsible decisions*.

(p. 117)

A fundamental element to achieve this purpose is the creation of an adequate link between teacher and student.

#### 3.3.1. The Relationship between the Teacher and the Student

The teacher can act as a secure base that provides stability and stimulates the children’s desire to learn based on the bond established with them. [44] agrees with [45] in considering that “*the basis of all learning processes is the affective bond between the child, the teacher and the other children in the class*” (p. 197). The same point of view is shared by [46] who also considers that the relationship that the teacher establishes with the children, especially if they present emotional needs, is key to being able to help them: “*it is precisely in the interpersonal relationship that these children can be helped the most. I would even say that it is the sine qua non condition*” (p. 26).

The conditions that, according to [45], should be present in the relationship between teacher and student to facilitate the process of personal growth are: the teacher’s authenticity, unconditional positive regard for the student and empathic understanding.

##### The Authenticity of the Teacher

A teacher educates through the values with which he/she is seen to live rather than through what he/she expresses in words. If he wants to have a positive influence on students, his discourse must be coherent with the way he acts; that is, he must be an example of what he preaches. [47] adds that “*when the teacher (…) shows himself in the relationship, without hiding behind a psychological and professional mask, there is a greater probability that the person (…) will grow constructively*” (p. 36).

##### Unconditional Positive Regard for the Student

The teacher must demonstrate a warm acceptance of the student and a real concern for him/her as a person, regardless of his/her behavior. Acceptance of the student as a person, although his/her behavior may be questioned, is a necessary condition for the student to feel safe in the relationship and to be able to communicate his/her feelings as he/she experiences them. In this sense, [46] also agrees with this premise when he expresses that the professional working with the child has to accept him/her as the person he/she is: “*the person is always loved and accepted (…) the behavior is not tolerated if it harms him/her or others (…) The being is always accepted*” (p. 27). As [48] also state, “*supportive relationships with students that bring unconditional positive regard can promote healing and growth*” (p. 139).

##### Empathic Understanding

This condition implies a deep understanding of the other person, beyond words. In the educational center, the teacher must not only listen to the student, but must also feel his/her inner world “*as if it were his/her own, but without ever losing the quality of “as if*” [45] (p. 250). According to [47], “*when a person feels listened to empathically, he/she comes to understand more accurately the flow of his/her own experiences*” (p. 37), a necessary aspect to give meaning to the experience one is going through and, therefore, to facilitate the resilient process.

#### 3.3.2. Classroom and School Climate

Educational centers create an institutional climate that can promote vulnerability or, on the contrary, act as a resilience-promoting factor. In classrooms, a key element is the way teachers are and how they relate to their students.

In addition to the relationship between teacher and students, the pedagogical strategies used, which can promote the establishment of positive relationships among peers, and even the physical setting of the space, are also very important in the creation of a good classroom climate. In the words of [48]: “*the classroom environment needs to be a place of comfort and safety. The teacher can foster a caring community where peers offer mutual support, as well as modify the physical environment*” (p. 138). Regarding this last element, [49] adds that “*emotional safety is often linked to a sense of belonging*” (p. 98) and points to the importance of children participating in the setting of the spaces they inhabit in order to personalize them and make them feel their own. According to [44], the school environment should provide a secure base “*sufficient for the child to use the resources of his intelligence to learn (…) instead of using all his energy defending himself*” (p. 221).

**Figure 2:**
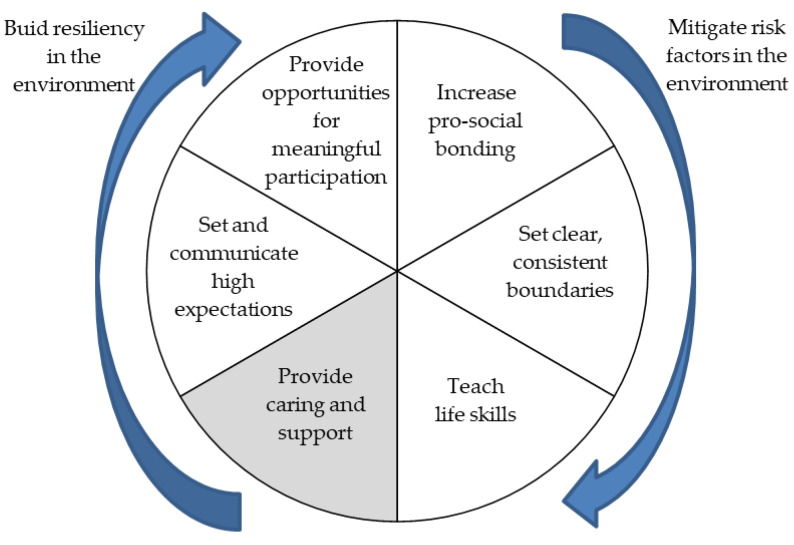
The Resiliency Wheel. Adapted from [20].

Ref. [20] are the authors of the Resiliency Wheel (Figure 2), a tool applicable to educational centers with the aim of creating resilience-promoting environments.

The different factors that are part of this tool can be described as follows:

##### Increase Pro-Social Bonding

This factor implies fostering positive interaction between the different members of the educational community: between teachers and students, between students and each other, and also between families and the school in general.

In the classroom, opportunities and time must be given to build relationships. A variety of methodologies should be used, promoting those that facilitate a high level of interaction, the establishment of helping relationships and the involvement of families. Bonding with peers enhances the group feeling. The organization of activities through cooperative learning or interactive groups, for example, favors the establishment of this type of relationship. [20] also mention the possibility of offering students a variety of activities before, during and after school hours that may appeal to children with different interests and motivations. All these activities can also promote bonding among the children themselves, and between them and other adults, such as monitors.

In the most disadvantaged contexts, these activities can be highly protective since, as the authors state, “*children with strong positive attachments are much less likely to engage in risky behaviors than those without*” [20] (p. 32).

##### Set Clear, Consistent Boundaries

Schools should develop coherent and consensual rules of coexistence, and make explicit the limits and behavioral expectations expected of their members. Children who do not have clear limits tend to be guided by behaviors promoted by their peers [20], especially by those who have greater leadership and who are not always the most favorable to them. It is important that students participate in the establishment of rules, limits and the determination of the consequences of not respecting them. Their participation will increase understanding and acceptance.

##### Teach Life Skills

Teaching to learn, to think, to know oneself, to communicate effectively, to resolve conflicts, to cooperate, to make consensual decisions, to establish shared goals… These skills, which must serve beyond the school environment, should not be taught in isolation, but must be included in the curriculum in a cross-cutting and contextualized manner. If students perceive that what they are learning is useful for life, they will be more interested in what they are being taught and will feel more connected to the institution. [20] mention cooperative learning as a way of acquiring skills to relate, express opinions, establish goals and make decisions in an integrated manner in activities.

##### Provide Caring and Support

According to the authors, this is the most fundamental aspect to help build resilience. Giving affection and support implies taking each child into account, recognizing him/her as an individual, knowing his/her interests, encouraging him/her to develop his/her abilities, detecting and making him/her aware of his/her strengths, and showing understanding and willingness to help him/her in the face of difficulties.

##### Set and Communicate High Expectations

It is important that “*expectations are both high and realistic in order to act as effective motivators”* [20] (p. 33). To this end, it is necessary to know each student well and to be able to give him/her the most personalized attention possible. The teacher has to make a self-reflection on the ways (conscious or not) how he/she transmits those expectations, taking care of the verbal and non-verbal language (gestures, looks, silences…). A varied methodology should be used and a variety of activities and evaluation tools should be offered that take into account different learning styles and ways of presenting information. This facilitates the learning of each child and favors that each child can have successful experiences, feel capable and can build a positive self-image.

##### Provide Opportunities for Meaningful Participation

Participation channels should be established so that students and their families have greater opportunities to make decisions that affect the functioning of schools and what is done in the classroom. Center and classroom projects, interactive groups, school mediation programs, cooperative learning, peer tutoring, among others, offer the possibility for students and/or families to participate in decisions that affect school organization. The participation of students and families in educational centers is a highly developed and successful aspect, for example, in schools that have been established as learning communities.

## 4. Conclusions

Throughout our life cycle, all of us will have to face different adversities, but we know that we have the capacity to activate a resilient process that will allow us to get through the situation and emerge stronger, so that we can continue to project ourselves into the future with greater self-confidence and greater trust in our possibilities. To make this possible, in addition to will and personal commitment, we must know and take advantage of our strengths and identify and make available resources in our environment that can be useful to us, allowing ourselves to be helped when necessary.

The educational center is a privileged place to promote resilience in children, especially in those who have suffered or are living through unfavorable experiences that affect them negatively on an emotional level and that can push them to develop difficulties in their relationship with themselves and with others. Teachers must favor resilient processes in students. To achieve this, they will have to bond with them, accompany them and build a relationship that makes them feel accepted and valued. They should also make use of pedagogical resources and strategies that contribute to promoting safe and resilient environments.

The ability to develop resilient processes should be part of the tools that students learn during their schooling. Making educational centers safe spaces that promote resilience is necessary and possible.

## Figures and Tables

**Table 1:** Documentary search criteria.

Database	Selection Criteria
Dialnet Plus	- Location of keywords: throughout the document. - Language: Spanish, English, Catalan. - The field of study is not limited. - Year of publication: 2000–2022.
WoS	- Location keywords: title. - Language: English and Spanish. - Limited by field of study. - Year of publication: 2012–2022.
Scopus	- Location: title, abstract, keywords. - Language: English and Spanish. - Limited by field of study. - Year of publication: 1993–2022.

Note: Own elaboration.

**Table 2:** Search equations.

Database	Search Equations	Results	Date
Dialnet Plus	(resiliencia OR resiliente) AND (escuel* OR escolar OR colegi* OR “centr* educativ*”) AND (emocional* OR conduct*) NOT (universidad OR adultos)	68	9 April 2022
WoS	(resilience OR resilient OR resiliency) AND school AND (emotional OR behavioral) NOT (adolescen* OR teen* OR high OR college OR university OR adult)	357	9 April 2022
Scopus	(resilience OR resilient OR resiliency) AND school AND (emotional OR behavioral) AND NOT (adolescen* OR teen* OR high OR college OR university OR adult)	363	9 April 2022
	Total	788	9 April 2022

Note: Own elaboration. * truncation (operator).

**Table 3:** Characteristics of the selected documents.

Authors	Year	Title	Type
Cyrulnik, B.	2006	Los patitos feos. La resiliencia: una infancia infeliz no determina la vida.	Book
Fonagy, P.; et al.	1994	The Emanuel Miller Memorial Lecture 1992. The Theory and Practice of Resilience.	Article
Forés, A.; Grané, J.	2012	La resiliencia en entornos socioeducativos.	Book
García-Yepes, N.	2020	Papel del docente y de la escuela en el fortalecimiento de los proyectos de vida alternativos (PVA).	Article
Gil, G.E.	2010	Los procesos holísticos de resiliencia en el desarrollo de identidades autorreferenciadas en lesbianas, gays y bisexuales.	Thesis
Gil, G.E.	2010	La resiliencia: conceptos y modelos aplicables al entorno escolar.	Article
Grotberg, E.H.	1995	A guide to promoting resilience in children: strengthening the human spirit.	Article
Henderson, N.; Milstein, M.	2005	Resiliencia en la escuela.	Book
Honsinger, C.; Brown, M.H.	2019	Preparing Trauma-Sensitive Teachers: Strategies for Teacher Educators.	Article
Luthar, S.S.; et al.	2000	The construct of Resilience: A Critical Evaluation and Guidelines for Future Work.	Article
Manciaux, M.; et al.	2010	La resiliencia: estado de la cuestión.	Chapter
Masten, A.S.; Coatsworth, J.D.	1998	The Development of Competence in Favorable and Unfavorable Environments. Lessons from Research on Successful Children.	Article
Puig, G.; Rubio, J.L.	2011	Manual de resiliencia aplicada.	Book
Richardson, G.E.	2002	The metatheory of Resilience and Resiliency.	Article
Ruiz-Román, C.; et al.	2020	Evolución y nuevas perspectivas del concepto de resiliencia: de lo individual a los contextos y a las relaciones socioeducativas.	Article
Rygaard, N.P.	2008	El niño abandonado. Guía para el tratamiento de los trastornos de apego.	Book
Salvo, S.; et al.	2017	¿La promoción de la resiliencia en la escuela puede contribuir con la política pública de salud?	Article
Theis, A.	2010	La resiliencia en la literatura científica.	Chapter
Villalba, C.	2003	El concepto de resiliencia. Aplicaciones en la intervención social.	Article

Note: Own elaboration from [16,17,18,19,20,21,22,23,24,25,26,27,28,29,30,31,32,33,34].

**Table 4:** Protective factors.

Authors	Protective Factors Specific to the Individual	Protective Factors Present in the Family and the Environment
Werner and Smith (1982, 1992)	Be female, physically strong, goal-oriented, adaptable, tolerant, a good communicator, socially responsible and have high self-esteem.	Have a good supportive environment within and outside the family.
Rutter (1979, 1985)	Being female, having a good temperament, self-control, perceived self-efficacy and planning skills.	Have a close, warm and stable personal relationship with at least one adult and a positive school climate.
Garmezy, Masten and Tellegen (1984); Garmezy (1991)	Temperament and personality attributes: having high expectations, reflective ability, good cognitive skills, positive outlook, high self-esteem, internal locus of control, self-discipline, problem-solving skills, critical thinking skills and sense of humor.	Family: good family cohesion that provides affection, presence of a relative other than the parents (grandparent…) who assumes the parental role in their absence or in case of relationship problems between them. Social support: availability of an adult who assumes the parental role if necessary, presence of a teacher who is interested in the child, and an organization or institution that provides support.
Kumpfer and Hopkins (1993)	Have good intellectual competence, capacity for introspection, high self-esteem, sense of direction or mission, be an empathetic and persevering person.	The authors make reference to the fact that, in the presence of the right conditions, the environment can promote resilience.
Benson (1997)	Internal developmental values: having educational commitment (internal motivation to learn), positive values (being caring, honest, responsible and upright), and social competence and positive identity (high self-esteem, internal locus of control and problem-solving skills).	External developmental values: receiving support (family, neighbors, school), knowing limits and expectations, and finding a constructive use of time.
Grotberg (1995, 2003)	I am: a person most people like, generally calm and well-disposed, someone who plans for the future and achieves what he or she sets out to do, a person who respects himself or herself and others. I can: generate new ideas or new ways of doing things; see a task through to completion; find humor in life and use it to reduce tension; express thoughts and feelings in communicating with others; resolve conflicts in different areas.	I have: a stable family and social environment; one or more people within my family group whom I can trust and who love me unconditionally; one or more people outside my family environment whom I can fully trust; limits on my behavior; people who encourage me to be independent; good role models; access to the health, education, security and social services I need.
Ungar (2003)	Self and interpersonal characteristics: having good intellectual and physical skills, a sense of self-efficacy, introspection, a positive self-image, high self-esteem, goals and aspirations, a sense of humor, creativity, empathy, self-expression, assertiveness, initiative, a sense of morality and commitment to values, and knowing how to maintain a social network by establishing meaningful relationships with others.	Family characteristics: having a quality upbringing and education, a flexible environment in which emotions are expressed, low levels of family conflict, and sufficient economic resources. Environmental and sociocultural characteristics: being socially included in a safe environment, having access to educational and leisure resources, perceiving social support, and membership in organizations.

Note: Adapted from [22,23,24,25,26].

**Table 5:** Pillars of resilience.

Authors	Pillars of Resilience
Wolin and Wolin (1993)	- Introspection. - Independence. - Interaction. - Initiative. - Creativity. - Moral awareness. - Sense of humor.
Rojas (2011)	- Personal executive functions. - Internal control center. - Healthy self-esteem. - Tendency to perceive positively. - Motives that give meaning to one’s life. - Rewarding affective connections.
Santos (2015)	- Introspection. - Motivation. - Emotional self-regulation. - Emotional independence and autonomy. - Self-confidence. - Ability to relate and establish mature emotional bonds. - Positive attitude and optimism. - Sense of humor and creativity. - Collaboration and commitment. - Ethics and coherence based on morals.
Linares (2017)	- Autonomy. - Problem solving. - Social skills. - Future purpose.

Note: Own elaboration from [27,28,29,30].

## Data Availability

Not applicable.
